# Two cases of rt-PA with dual antiplatelet therapies with capsular warning syndrome

**DOI:** 10.1097/MD.0000000000024698

**Published:** 2021-03-05

**Authors:** Xiaofan Xue, Heng Zhou, Lichun Zhou

**Affiliations:** a Department of Neurology, Beijing Chaoyang Hospital, Capital Medical University, Beijing, China; b MasGeneral Institute for Neurodegenerative Disease, Department of Neurology, Massachusetts General Hospital, Charlestown, Boston, MA, USA.

**Keywords:** Antiplatelet therapy, capsular warning syndrome, case report, recombinant tissue plasminogen activator, stroke warning syndrome

## Abstract

**Rationale:**

Capsular warning syndrome (CWS) is a term to describe stereotyped lacunar transient ischemic attacks (TIAs). Patients with CWS are at high risk of developing completed stroke. However, the exact pathophysiology of CWS is still unclear, and there is no conclusive clinical strategy for CWS patients.

**Patient symptoms:**

Two cases of middle-aged men with hypertension, hyperlipidemia, and diabetes mellitus presented with fluctuating right-sided weakness, numbness, and dysarthria.

**Diagnoses:**

These two patients were diagnosed with CWS.

**Interventions:**

Recombinant tissue plasminogen activator (rt-PA) intravenous thrombolysis (0.9 mg/kg) was administered first and treated with aspirin (100 mg) and clopidogrel (75 mg) after 24 h of rt-PA for 21 days following by aspirin (100 mg) alone.

**Outcomes:**

Both cases got favorable clinical outcomes of somatic symptoms. In addition, diffusion-weighted imaging (DWI or DW-MRI) showed that ischemic injury disappeared in case 1 while maintained within a reasonable range in case 2.

**Lessons:**

Early recognition and rt-PA/dual antiplatelet treatment may be an effective strategy for patients with CWS.

## 1 Introduction

Capsular warning syndrome (CWS) is a term used to describe the phenomenon of repeated transient ischemic attacks (TIAs) represented by sensory and/or motor symptoms (≥ three times within 24 h) with at least two of the face, arm, and leg, without cortical signs (aphasia, apraxia, and agnosia).^[1]^ The patients with CWS have a much higher risk for developing early stroke than those patients with a previous TIA.^[2,3]^ CWS is also considered a rare subtype of TIA and has often been misdiagnosed as a conventional TIA, which results in delayed therapy and the deterioration of patient's condition. The most common cause of CWS is small perforating artery disease which induces focal ischemic attacks. However, there is still uncertainty about the therapeutic strategies that are effective for CWS. To the best of our knowledge, there were some cases of CWS treated with rt-PA administration and dual antiplatelet therapy. Here, we reported two cases of middle-aged men with CWS who were treated with recombinant tissue plasminogen activator (rt-PA) administration followed by 21-days dual antiplatelet therapy, that resulted in somatic symptoms improvements and resolution of the patient's TIAs.

## 2 Case representation

**Figure 1:**
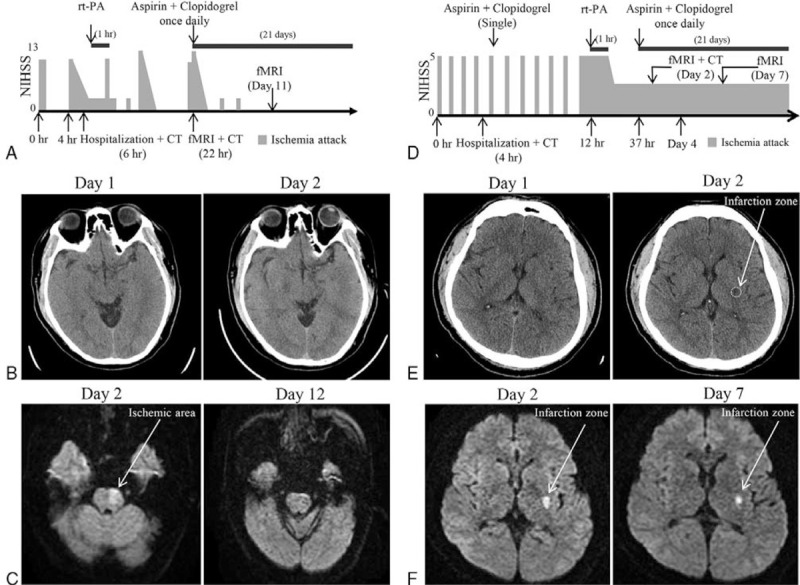
The treatment of the patients. (A) Schematic representation of the clinical course for case 1. (B) Brain CT of case 1 on the day 1 and 2 were normal. (C) DWI showed hyperintense area in the left pontine after 18 h of rt-PA treatment, and the DWI was normal after 11 days of rt-PA. (D) Schematic representation of the clinical course for case 2. (E) Brain CT of case 2 on the day 1 was normal, the Brain CT on the day 2 showed hyperintense area in the left internal capsular after 24 h of rt-PA. (F) DWI showed hyperintense area in the left internal capsule after 24 h of rt-PA treatment, and DWI also showed the same after 7 days of rt-PA.

Case 1: A 49-year-old man showed fluctuating right-sided weakness, numbness, and dysarthria. The initial episode (Day 0) lasted 60 min before it resolved to normal and the second one lasted for 120 min. Neurological examination revealed slight speech difficulty, slight weakness of right-sided limbs, positive Babinski sign on the same side. His National Institute of Health Stroke Scale (NIHSS) was 10 points, with high blood pressure, triglyceride (8.97 mmol/L), random blood sucrose (15.18 mmol/L) when he was admitted to the hospital (Table 1). He was administered rt-PA (0.9 mg/kg) for 1 h after the second TIA episode. He still experienced five short-duration (3–10 min) stereotypical TIAs within 48 h after rt-PA with fluctuating NIHSS from 2 to 13 points (Fig. 1A). The cranial computerized tomography (CT) scan did not show any abnormality before or after rt-PA treatment (Fig. 1B). Diffusion-weighted magnetic resonance imaging (DWI or DW-MRI) was performed 1 day after the rt-PA that revealed a hyperintense signal involving in the left pontine which disappeared at day 12 during the second DWI (Fig. 1C). He was daily treated with aspirin (100 mg) and clopidogrel (75 mg) after 24 h of rt-PA for 21 days following by aspirin (100 mg) alone. No further episodes were observed after 2 days of rt-PA. Neurological examination was otherwise normal with a good recovery after 6 months follow up.

**Table 1:** Clinical characteristics of the 2 cases.

Item	Case 1	Case 2
Age (years)	49	45
Sex	Male	Male
Vascular risk factors	HLP, DM, SMK, HT	HLP, DM, SMK, HT
TIA episodes before rt-PA	1	10
Symptoms	Right-sided weakness, numbness and dysarthria	Right-sided weakness, numbness, right-sided facial droop and dysarthria
Blood Pressure (mmHg)	145/96	153/111
NIHSS at admission	10	0 (ABCD2 score = 4)
NIHSS at discharge	0	2
mRS at admission	0	0
mRS at discharge	0	1

Case 2: A 45-year-old man presented with fluctuating right-sided weakness and numbness, right-sided facial droop and dysarthria. The initial episode (day 0) lasted 10 min before returning to normal, which occurred further three times with same symptoms and similar duration. While, there were no signs after the body examination at the time of arrival to hospital. He had a medical history of hyperlipidemia and he was a smoker for 10 years. Blood pressure was 153/111 mm Hg. He had type 2 diabetes and sleep apnea–hypopnea syndrome (Table 1). ABCD2 score was 4. He was diagnosed as high-risk TIA and administered dual antiplatelet (100 mg aspirin and 75 mg clopidogrel). However, the same TIA episode occurred again six times with 4- to 10-min duration within 12 h in which each episode was sudden at onset and resolved completely. The NIHSS score was 5 during each episode. His symptoms improve and lasted for over 30 min at the 11th TIA episode. Thus, he received rt-PA administration for 1 h. Symptoms gradually improved, and NIHSS score decreased to 2 after rt-PA treatment. Dual antithrombotic and atorvastatin treatment were provided after day 2 (Fig. 1D). The cranial CT scan did not show abnormality at the time of hospitalization, but a hyperintense area was found in the left internal capsule during the second scan at day 2 (Fig. 1E), which was confirmed by DWI at day 2 while the size of hyperintense area became smaller at the second DWI test at day 7 (Fig. 1F). No further TIA episode was observed. This patient also daily received dual antiplatelet for 21 days followed by only aspirin. After 6 months follow up, the patient showed a good recovery.

## 3 Discussion

CWS, a rare clinical syndrome, is caused by recurrent stereotyped TIAs with complete recovery between episodes.^[1]^ Lots of patients have infarctions in the internal capsule, and the others experience it in different regions including pontine and callosal.^[4,5]^ Thus, some researchers extend this notion to “Vascular/Stroke Warning Syndrome”.^[5,6]^ Here, we reported two cases presenting as fluctuating right-sided weakness, numbness and dysarthria. One had infarctions in the left pontine and the other had infarctions in the left internal capsule. Both patients got favorable outcomes after rt-PA intravenous thrombolysis and followed by 21-days dual antiplatelet therapy.

While the exact pathophysiology of CWS is still unclear, existing hypotheses include hemodynamic impairment, vasospasm, artery-to-artery embolism, and periinfarct depolarization.^[7]^ However, it is still unclear about how to block the development of CWS to completed stroke. Although various antithrombotic treatments such as antiplatelets, heparin, and thrombolytics have been tried to halt this process, it is still uncertain if any of these therapies are effective.^[8]^ In previous reports, some patients got favorable recovery after rt-PA treatment in acute phase loading with clopidogrel or long term of multiple antiplatelet therapy.^[4,5,9]^

In this report, both patients had hypertension, hyperlipidemia and diabetes mellitus, which are high risk factors of small vessel disease including the stenosis of small vessels and hemodynamic impairment. They received rt-PA treatment in acute phase and followed by 21-days dual antiplatelet therapy. The NIHSS score of case 1 was dramatically reduced (from 10 to 0) after rt-PA treatment, but the patient still experienced five TIAs later, which indicated that rt-PA can effectively attenuate the symptom but cannot totally block the following TIAs. The results of magnetic resonance images (MRI) indicated that the subsequent TIAs did not produce a permanent focal injury. Thus, case 1 got a total recovery under this clinical strategy. Similarly, the NIHSS score of case 2 also reduced from 5 to 2 after rt-PA treatment. However, the hyperintense area in the left internal capsular of brain MRI did not totally disappear. It could be explained by the fact that the number of TIAs before rt-PA treatment of case 2 was much higher than case 1, which also extended the time window of rt-PA treatment. Meanwhile, the reduced hyperintense area of MRI indicated that the rt-PA might partially rescue the ischemic penumbra. The other hypothesis should be the TIAs occurred in different brain regions in the two patients. It is worthy to use the focal ischemic model of animal in different brain regions with different time window to address these questions and the pharmacological mechanism in the future.^[10]^

Taken together, early recognition and therapy is critical for CWS therapy and complete stroke recovery. Our cases provide the possible clinical strategy of rt-PA administration with dual antiplatelet therapy for CWS. However more studies utilizing this strategy are needed.

## Acknowledgments

The authors appreciate the patients‘ cooperation sincerely. All authors read and approve the final manuscript and they have no competing interests.

## Author contributions

XFX and LCZ were responsible for data collection, overseeing the concept and design of the study. XFX, HZ and LCZ were responsible for paper writing.

**Investigation:** Xiaofan Xue.

**Writing – original draft:** Xiaofan Xue.

**Writing – review & editing:** Xiaofan Xue, Heng Zhou, Lichun Zhou.
